# Multiparametric Longitudinal Profiling of RCAS-tva-Induced PDGFB-Driven Experimental Glioma

**DOI:** 10.3390/brainsci12111426

**Published:** 2022-10-24

**Authors:** Hannes Becker, Salvador Castaneda-Vega, Kristin Patzwaldt, Justyna M. Przystal, Bianca Walter, Filippo C. Michelotti, Denis Canjuga, Marcos Tatagiba, Bernd Pichler, Susanne C. Beck, Eric C. Holland, Christian la Fougère, Ghazaleh Tabatabai

**Affiliations:** 1 Department of Neurology & Interdisciplinary Neuro-Oncology, Hertie Institute for Clinical Brain Research, Center for Neuro-Oncology, Comprehensive Cancer Center, University Hospital Tübingen, Eberhard Karls University Tubingen, 72072 Tubingen, Germany; 2 Department of Neurosurgery, University Hospital Tubingen, Eberhard Karls University Tubingen, 72072 Tubingen, Germany; 3 Werner Siemens Imaging Center, Department of Preclinical Imaging and Radiopharmacy, Eberhard Karls University Tuebingen, 72072 Tubingen, Germany; 4 Department of Nuclear Medicine and Clinical Molecular Imaging, Eberhard Karls University Tuebingen, 72072 Tubingen, Germany; 5 German Translational Cancer Consortium (DKTK), DKFZ Partner Site, 72072 Tubingen, Germany; 6 Cluster of Excellence iFIT (EXC 2180) “Image Guided and Functionally Instructed Tumor Therapies”, Eberhard Karls University, 72072 Tubingen, Germany; 7 Human Biology Division, Fred Hutchinson Cancer Research Center, Seattle, Washington, DC 98109, USA

**Keywords:** glioblastoma, RCAS-tva, rodent glioma models, metabolism, multiparametric PET/MR imaging, model characterization, PDGFB

## Abstract

Glioblastomas are incurable primary brain tumors harboring a heterogeneous landscape of genetic and metabolic alterations. Longitudinal imaging by MRI and [^18^F]FET-PET measurements enable us to visualize the features of evolving tumors in a dynamic manner. Yet, close-meshed longitudinal imaging time points for characterizing temporal and spatial metabolic alterations during tumor evolution in patients is not feasible because patients usually present with already established tumors. The replication-competent avian sarcoma-leukosis virus (RCAS)/tumor virus receptor-A (tva) system is a powerful preclinical glioma model offering a high grade of spatial and temporal control of somatic gene delivery in vivo. Consequently, here, we aimed at using MRI and [^18^F]FET-PET to identify typical neuroimaging characteristics of the platelet-derived growth factor B (PDGFB)-driven glioma model using the RCAS-tva system. Our study showed that this preclinical glioma model displays MRI and [^18^F]FET-PET features that highly resemble the corresponding established human disease, emphasizing the high translational relevance of this experimental model. Furthermore, our investigations unravel exponential growth dynamics and a model-specific tumor microenvironment, as assessed by histology and immunochemistry. Taken together, our study provides further insights into this preclinical model and advocates for the imaging-stratified design of preclinical therapeutic interventions.

## 1. Introduction

A glioblastoma is one of the most aggressive primary tumors in the central nervous system. Despite multimodal therapy approaches, glioblastomas still have a devastating prognosis, with a median overall survival in the range of 1.5 years [1,2,3,4,5]. Standard of care therapy outside of clinical trials consists of maximal safe resection followed by a combination of radiation therapy, alkylating chemotherapy, and tumor-treating fields [1,3,6,7,8]. Still, tumor progression is inevitable in most patients. Re-resection can be considered if the whole contrast-enhancing tumor volume can be removed without new neurological symptoms [7,9,10]. Furthermore, alkylating chemotherapy or other systemic therapies are considered in interdisciplinary tumor boards [7,9,10,11].

Comprehensive genetic sequencing efforts over the last decades have revealed a complex network of mutations in human cancer entities [12]. Multi-omic approaches and extensive bioinformatic clustering analysis have enabled the definition of progressively sharper subsets of different cancer entities [13,14,15,16]. This scientific progress contributed to the better understanding of its biology and of potential treatment targets in some entities [14,17]. Furthermore, molecular profiling enables reverse translation approaches to design more accurate preclinical models [18,19]. In glioblastomas, genetic alterations are clustered into three subtypes: classical, proneuronal, and mesenchymal [14,16,20]. Besides genetical alterations, metabolic adaptation and the reprogramming of cancers cells have been defined as an additional hallmark of cancer [21,22]. In fact, immunosuppressive reshaping of the glioma-associated microenvironment by the release of oncometabolites and a distinct landscape of metabolomic alterations in human glioma have highlighted the importance of metabolic fine tuning for human glioma [23,24]. In vivo imaging using [^18^F]FET-PET is a strong diagnostic tool in neuro-oncology when assessing tumor metabolism on a molecular level and has become a standard tool in clinical neuroimaging [25]. [^18^F]FET-PET is an amino acid radiotracer that is taken up by proliferating tumor cells and has been associated with L-type amino acid transporters (LAT) [26]. PET/MRI plays an important clinical role in the evaluation of metabolically active regions and thereby in differentiating therapy-associated pseudoprogression from real tumor progression [7,25]. In particular, [^18^F]FET-PET improves sensitivity, specificity, and accuracy in differentiating glioma from non-neoplastic tissue [27,28,29]. [^18^F]FET-PET/MRI has a value in the differentiation between tumor progression and so-called pseudoprogression [30,31,32,33]. Furthermore, contrast-enhanced MRI can evaluate blood brain barrier (BBB) permeability with high sensitivity, and pseudoquantification can be performed [34,35]. Moreover, it has been correlated with tumor infiltration and associated with tumor vascularity, as well as progression-free survival [36,37].

**Figure 1:**
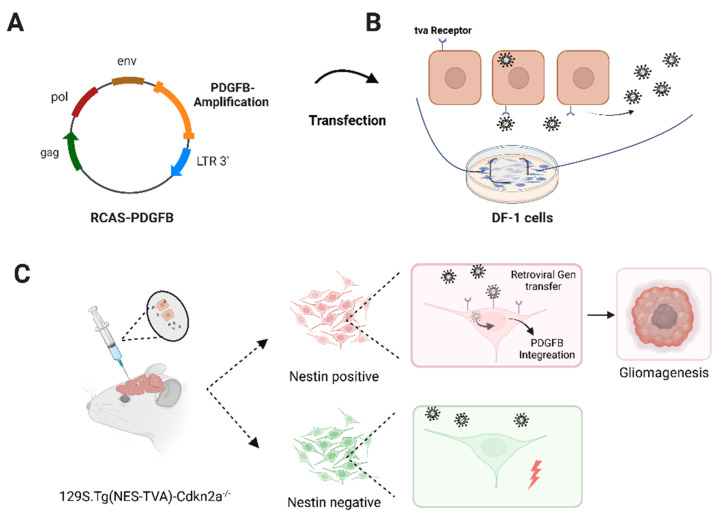
Schematic overview of the PDGFB-driven glioma mouse model using the RCAS-tva delivery system. (**A**) Schematic overview of the RCAS plasmid including a PDGFB amplification. This vector is transfected into DF-1 cells. (**B**) Viral replication takes place after cellular entry via tva receptor binding. (**C**) Implantation of 5 × 10^4^ transfected DF-1 cells into genetic engineered animals. Tissue-dependent expression of the tva receptor is ensured by expression of tva controlled by the nestin promoter in mice. This leads to the infection of only the nestin-positive cell population (red). Subsequently, the PDGFB amplification is integrated retrogradely into the host genome. Together with the systemic deletion of the cell cycle regulator Cdkn2a, intracerebral tumor formation occurs. Created with BioRender.com.

Preclinical models recapulating the genetical and metabolic alterations of human gliomas are urgently needed for dissecting potential vulnerabilities during glioma evolution in preclinical in vivo settings [38]. In this regard, somatic gene transfer systems that offer the spatial and temporal control of gene delivery are of high scientific and translational value [39,40]. One retrovirus-based system that displays these characteristics is the replication-competent avian sarcoma-leukosis virus (RCAS)/tumor virus receptor-A (tva) system [41]. As illustrated in Figure 1A, the RCAS virus offers a multicloning site for the insertion of genetic information with a maximum of 2.5 kilobase (kb), which is controlled by the viral LTR promoter [42]. Viral replication solely takes place in cells expressing the tva receptor, which is essential for viral entrance. Unlike mammalian cells, avian cells, such as the chicken fibroblast long-term cell line DF-1, naturally express the tva receptor (Figure 1B).

The establishment and breeding of immunocompetent, transgenic mice expressing the tva receptor under the control of tissue-specific promoters allows the orthotopic induction of diverse tumor entities using a broad range of oncogenic drivers (example shown in glioma setting in Figure 1C). For instance, mouse models using the RCAS-tva delivery system are available for glioma, ependymoma, pancreatic cancer, hepatic cancer, and ovarian cancer research [41,42,43,44,45]. Additionally, rodent crossbreeding offers large genetic combination possibilities. Examples are the implementation of luciferase-dependent in vivo imaging as well as the combination of existing gene editing systems, such as the widely used cre-lox or CRISPR-Cas9 editing systems for inducing chromosomal translocation or gene fusions [42,46,47,48].

A platelet-derived growth factor B (PDGFB)-driven glioma model using the RCAS-tva system has been used to test a wide range of novel therapeutic targets [49,50,51]. It comprises genetic features of the proneuronal glioma subtype by combining PDGFB amplification with the lack of cell cycle regulator Cdkn2a (Figure 1) [14,16,46,52,53].

The aim of the study was to investigate (i) the growth dynamics with a special focus on identifying neuroimaging characteristics of the PDGFB-driven glioma mouse model using clinically relevant imaging methods, i.e., preclinical [^18^F]FET-PET/MRI, and (ii) to study features of the glioma-associated microenvironment by immunohistochemistry.

## 2. Materials and Methods

### 2.1. DF-1 Cell Transfection

DF-1 cells were cultured in Dulbecco’s Modified Eagle’s Medium (DMEM) (ThermoFisher, Waltham, MA, USA) at 39 °C with 5% CO_2_ atmosphere [54]. Cells were seeded 24 h before transfection. An amount of 2.5 µg of the RCAS-PDGFB or RCAS-GFP plasmid was dissolved in 150 µL DMEM, together with 25 µL SuperFect^®^ Transfection Reagent (Qiagen, Venlo, The Netherlands), and incubated for 7 min at room temperature (RT) to form complexes [41,46]. Next, 1 mL of DMEM was added, and the solution was transferred to the seeded DF-1 cells and incubated for 3 h at 39 °C 5% CO_2_. Afterwards, cells were washed and cultured with DMEM. Transfection control was assessed by fluorescence microscopy. The GFP signal of RCAS-GFP-transfected DF-1 cells was evaluated starting at day 5 post transfection. Pictures were taken with an Axiovert 200M imaging system (Zeiss Microscopy, Oberkochen, Germany).

### 2.2. Transfected DF-1 Cell Implantation into Immunocompetent (129S.Tg(NES-TVA)-Cdkn2a^−/−^) Mice

Animal experiments were conducted in accordance with the local authorities and the German laws regulating the appropriate use of laboratory animals. Described procedures and experimental settings were approved by The Institute of Animal Welfare and the Veterinary Office at the University of Tubingen and the Regional Council Tuebingen. We used a PDGFB-driven glioma mouse model using the RCAS-tva somatic gene transfer delivery system as originally established and described by Hambardzumyan et al. [46]. Fifty thousand RCAS-PDGFB transfected DF-1 cells were implanted intracranially in the same manner as described previously [55,56,57].

In brief, adult mice (male and female) (129S.Tg(NES-TVA)-Cdkn2a^−/−^) [53] were anesthetized with a 3-component anaesthetic (fentanyl, midazolam, and medetomidine). Then, the anatomical injection site, the right striatum, was located using a stereotactic device (Stoelting, Wood Dale, IL, US). Next, 5 × 10^4^ transfected DF-1 cells resuspended in 1 × PBS, in a volume of 2 µL were injected into the mice using a Hamilton syringe (Hamilton Bonaduz AG, Bonaduz, Switzerland). Intracranially implanted mice were carefully monitored and longitudinally imaged as outlined in Section 2.3. Tumor-bearing mice were euthanized at the appearance of moderate clinical signs, which were regularly assessed according to a predefined, previously described scoring system shown in detail in Supplementary Table S1 [57,58,59,60].

### 2.3. MRI and [^18^F]FET-PET Measurements

PET and MRI acquisitions were performed longitudinally over a period of 42 days in tumor-bearing mice (*n* = 8), starting 6 days after implantation as outlined in Section 3.1. Isoflurane with a 2.5% induction and 1–1.5% maintenance using room air at a flow rate of 1.5 L/min was used as an anesthetic during the measurements. A constant body temperature of 37 ± 0.5 °C was maintained throughout the acquisitions, using temperature regulated bed systems for MRI (Bruker Biospin, Ettlingen, Germany) and PET (Medres, Cologne, Germany).

MRI scans were performed using a 7T Clinscan small-animal MR scanner equipped with a whole-body transmitter coil and a volume coil that completely covered the mouse head (Bruker Biospin, Ettlingen, Germany). Respiratory rate and gating for MRI sequences were performed using a breathing pad. T2 weighted images (T2W) were acquired using a 2D-spoiled turbo RARE spin echo sequence (256 × 256 matrix, 20 × 20 mm^2^ field of view (FOV), repetition time (TR) = 2500 ms, echo time (TE) = 33 ms, slice thickness = 0.7 mm, 18 slices, averages = 2). T1-weighted images were acquired with increasing flip angles using the following parameters: 129 × 129 matrix, 25 × 25 mm^2^ field of view (FOV), repetition time (TR) = 10 ms, echo time (TE) = 1.34 ms, slice thickness = 0.2 mm, flip angles = [2,9,27], 80 slices, averages = 2. For contrast enhancement, T1-weighted images were measured using the above-mentioned parameters approximately 4 min after the intravenous contrast agent injection of Gadobutrol (Gadovist^®^ 1 mmol/mL, Bayer Schering Pharma, Berlin, Germany) diluted to a concentration of 0.2 mmol/mL at a dosage of 25 mmol per kg of body weight. Relaxometry T1-maps were calculated by linearly fitting the T1-weighted images voxel-wise, as previously described using Matlab (Matlab 2013b, The MathWorks, Natick, MA, USA) [34,61]. To further validate the T1-maps and quantify gadolinium concentrations in the brain, we acquired T1-weighted images of a phantom containing linearly increasing concentrations of Gadobutrol. PET acquisitions were performed using brain-dedicated beds using an Inveon small-animal PET scanner (Siemens Healthineers, Erlangen, Germany). A bolus of 11.6 ± 0.74 MBq of O-(2-[^18^F]fluoroethyl)-L-tyrosine ([^18^F]FET) was injected in the catheterized tail vein and measured using PET through a dynamic acquisition of 50-min in list-mode. The data were histogrammed in a 10-min time frame and reconstructed using an iterative ordered subset expectation maximization (OSEM3D) algorithm. For attenuation correction, a 10-min transmission acquisition using a 57Co source was acquired for every PET measurement.

### 2.4. MR Image Analysis

The acquired PET and MRI images were realigned and co-registered using Pmod Software v3.2 (Bruker Biospin, Ettlingen, Germany). Regions of interest (ROI) were drawn in agreement with two experienced neuroimaging scientists using the contrast enhanced T1-weighted images. The ROI masks were used to extract data from the T1-relaxometry maps and dynamic PET image data.

### 2.5. Scoring of Experimental Animals and SMA560&VM/Dk Mice

After transfected DF-1 cell implantation, the animals were closely examined, and the clinical and neurological symptoms were evaluated according to a well-established scoring scheme (Supplementary Table S1) [58]. The endpoint of the experiments was set at a moderate physical burden. As soon as clinical endpoints were met, the experimental animals were euthanized as defined by the responsible governmental authority (Regional council Tuebingen).

Described IHC images in Section 3.3 were taken from either transgenic mice after DF-1 cell implantation or immunocompetent mice of the orthotopic SMA560/VM-Dk model [62]. In brief, 5 × 10^3^ SMA560 cells were implanted in the same manner as described in Section 2.2. Mice were part of an experiment previously published [57]. Shown mice belonged to the control group, which received an isotype control antibody (MOPC-21) intraperitoneally once per week (30 mg/kg). The isotype control was provided by Roche Diagnostics (Penzberg, Germany). Treatment started at day 7 post-tumor cell implantation. The experiment was closed at day 18 post-tumor cell implantation.

### 2.6. Immunohistochemistry of Murine Tumor Samples

The following antibodies were used: CD3, CD4, CD8, CD11b, CD19, CD20, CD45R, CD163, Ki67, NCRI (Abcam, Cambridge, UK), CD204 (ThermoFisher, Waltham, MA, USA), CD31 (BD Biosciences, Heidelberg, Germany), PD1, and PD-L1 (ProSci, Poway, CA, USA). After reaching the predefined experimental endpoint, animals were perfused with ice cold PBS, and brains were snapped frozen. Then, 8 µm thick sections were cut using a Leica CM3050S cryostat (Leica, Wetzler, Germany). Brain slices were stored at −80 °C.

First, brain sections were dried at room temperature for 10 min, and fixation was performed either with ice-cold acetone at −20 °C for 10 min or 4% PFA for 15 min. Endogenous peroxidase activity was blocked by Bloxall (Vector Laboratories, Peterborough, UK). Next, brain sections were incubated with 10% bovine serum albumin (BSA) in PBS-Tween 0.3% for 1 h at RT. The primary antibody was incubated overnight at 4 °C. After several washing steps with PBS, slides were incubated for 1 h at RT, with respective biotinylated secondary antibodies diluted in 2% BSA PBS-Tween 0.05%. Vectastain^®^ ABC Kit as a signal amplifier and Vector NovaRED (Vector Laboratories) as a detection kit were used. Counterstaining with Hematoxilin (Sigma-Aldrich, St. Louis, MO, USA) was performed. Finally, slides were dehydrated and were mounted in DPX medium (VWR, Radnor, PA, USA). Stained tissue sections were analysed under a Carl Zeiss Axioplan2 Imaging brightfield microscope (Zeiss Microscopy, Oberkochen, Germany) with the Axio Vision 4.0 software.

### 2.7. Statistics

In vivo symptom-free survival was evaluated with Kaplan–Meier survival fractions, *p* values were generated, and the Log-rank test (Mantel–Cox) was performed. Additionally, the Tukey-Kramer post hoc test was used. Error bars represent standard deviation (SD). Pearson correlations were calculated using MATLAB (R2021b, The MathWorks, Inc., Natick, MA, USA).

Growth curves of contrast-enhancing regions and gadolinium- and FET-uptake were analysed by GraphPad Prism 9 (GraphPad Software, San Diego, CA, USA) and visualized with Adobe Illustrator 2022 (Adobe, San José, CA, USA).

## 3. Results

### 3.1. Implantation of RCAS-PDGFB Transfected DF-1 Cells and Glioma Formation In Vivo

**Figure 2:**
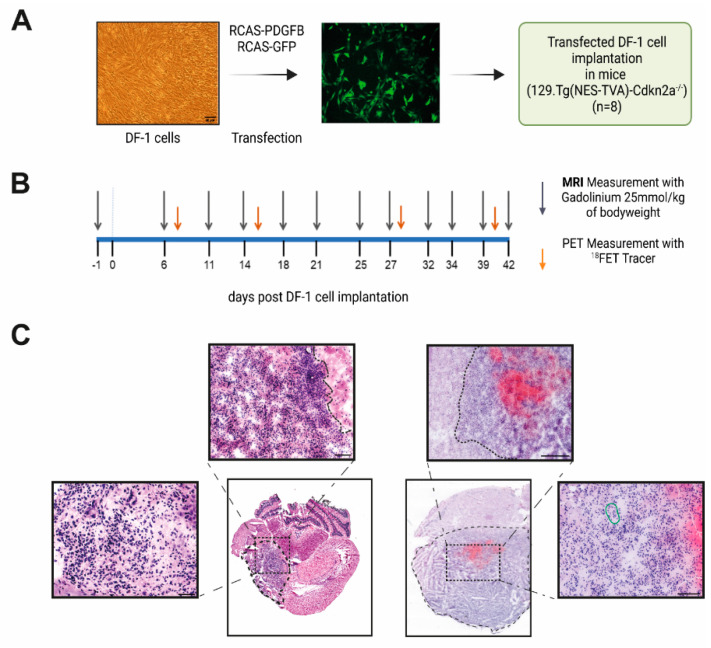
Longitudinal MRI-FET-PET imaging study and H&E staining pattern. (**A**) Schematic overview of experimental design. Brightfield image shows transfected DF-1 cells. Scale bar is 50 µm. Representative picture of DF-1 cells transfected with RCAS-GFP plasmid as transfection control. (**B**) Imaging schedule: day 0 represents the day of implantation of transfected DF-1 cells. (**C**) Representative images of two animals showing glioma formation. Typical aspects of high-grade glioma, such as infiltrating growth behavior, neovascularization (highlighted in green), and cell atypia, are visible. Boundaries of the tumor core area are highlighted with a dotted line. Scale bars are 50 and 100 µm. (**A**,**B**) were created with BioRender.com.

**Figure 3:**
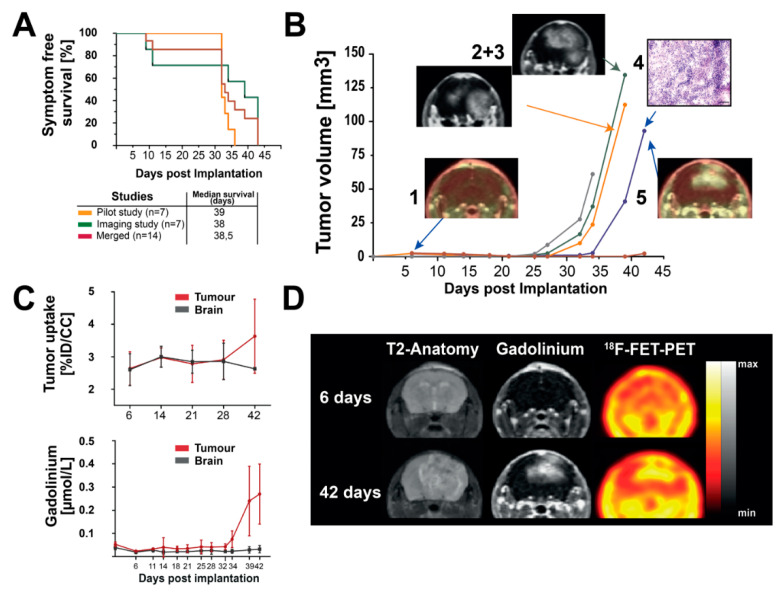
(**A**): Kaplan–Meier plots of symptom-free survival of the longitudinal observational study with and without MRI and FET-PET measurements (yellow and green curve). The red curve represents entire animal studies. No statistically significant differences in Log-Rank test and Tukey–Kramer post hoc test were observed. (**B**) Longitudinal tumor volume development: (1) representative FET-PET image day 7; (2) and (3) representative MRI images of day 39 PTI (post tumor initiation) of two animals showing glioma-like gadolinium enhancement in the final stage of the disease; (4) representative H&E staining showing glioma-like histologic features in the region of the previously occurred gadolinium enhancement (scale bar is 50 μm); (5) representative FET-PET image, showing FET uptake in tumor regions at day 42 PTI. (**C**) (1) Analysis of [^18^F]FET uptake in five representative imaged animals. (2) Analysis of longitudinal gadolinium uptake in tumor regions and normal brain regions. Error bars represent standard deviation (SD). (**D**): Longitudinal MRI and FET-PET images of one representative animal. Color bars as the reference of gadolinium and [^18^F]FET uptake are shown.

First, we generated murine glioma in vivo after the implantation of RCAS-PDGFB transfected DF-1 cells in experimental mice in two independent animal experiments with and without longitudinal imaging. Therefore, as illustrated in Figure 1 and Figure 2A, DF-1 cells were transfected with RCAS-PDGFB [46]. Transfection was controlled by a GFP-labelled transfection control (RCAS-GFP), showing an enhanced GFP signal after plasmid transfection (see Figure 2A). Next, 5 × 10^4^ transfected DF-1 cells were implanted into the right striatum of the mice (day 0). For longitudinal MRI and FET-PET imaging, mice received baseline measurement one day before cell implantation (see Figure 2B). Animals received cerebral MRI measurements twice per week (in total, 12 measurements) and an additional FET-PET once per week depending on the availability of the FET tracer, as visualized in Figure 2B. Untreated symptom-free survival was assessed according to the experimental endpoint, as outlined in material/methods and Supplementary Table S1. Median symptom-free survival time in the imaging animal group was 38 days; the independent implantation of transfected DF-1 cells in seven additional animals revealed a median symptom-free survival of 39 days (see Figure 3A).

**Figure 4:**
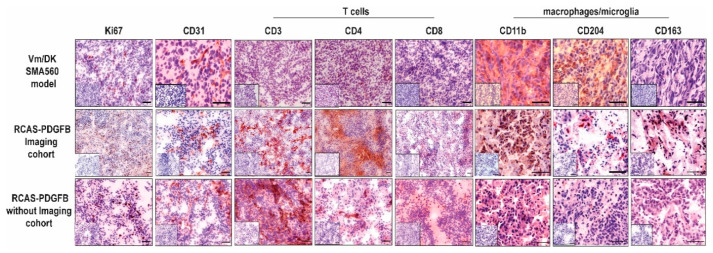
Immunohistochemical comparison of established T cell, microglia/macrophages, and vascularisation markers as indicated above. Representative IHC staining patterns of tumor tissues of either VM/Dk mice treated with an isotype control antibody (MOPC-21), as described in Przystal et al., or transgenic mice after the implantation of RCAS-PDGFB transfected DF1 cells. Small inserts show staining control without the application of primary antibody. Scale bars are 100 µm.

We performed Hematoxylin and Eosin (H&E) staining for the histological confirmation of tumor formation in post-experimental brain tissue. Additionally, immunohistochemistry for the proliferation marker Ki67 as well as endothelial marker CD31 were executed (see Figure 4, column 1–2). Both experiments revealed extensive tumor formation with glioma-type histological characteristics [20]. We observed infiltrative growth behavior, a high density of strong basophilic stained tumor cells, a high grade of neovascularization, and an extended necrosis area (as illustrated in Figure 2C). This infiltrative growth was more pronounced compared to the SMA560-glioma (see Supplementary Figure S1). Proliferation marker Ki67 was largely present in tumor regions and was good comparable to the staining patterns in untreated VM-Dk mice (see Figure 4, column 1). Staining against CD31 showed large vessel formation in good accordance with the PDGFB amplification as the tumor driver mutation (see Figure 4, column 2). CD31 signal was present in the widely used orthotopic SMA560/VM-Dk glioma mouse model as well. Interestingly, tumor volume expanded over the adjacent brain regions, infiltrating into the adjacent temporal and parietal lobe. Taken together, we could detect robust tumor formation, which could be confirmed by histology, displaying high-grade glioma-like histological features.

### 3.2. Longitudinal MR-Imaging Reveals Exponential Growth Dynamics and Late FET Uptake

Acquired imaging data, a total of 12 MRI measurements and 4 FET-PET measurements, were co-registered, and the longitudinal course was analysed. As shown in Figure 3B, the longitudinal volumetry of the contrast agent-enhanced regions revealed tumor-specific signals starting at day 25 until 27 after the implantation of transfected DF-1 cells. First, the contrast agent-enhancing lesions were detected in the region of cell implantation and were expanded and showed increasing accumulations in an exponential fashion. Irregular and diffuse gadolinium uptake, similar to the radiological behavior of human high-grade glioma, was detected. At the final phase of acquired measurements, gadolinium uptake was detected even contralateral to the implantation side, infiltrating the corpus callosum and the contralateral parietal lobe (see Figure 3B(2 + 3),D). In the T2-weighted images, a large signal alteration was apparent in comparable localizations. Furthermore, in the region of the cell implantation, T2-alterations highly suspected for tumor necrosis were detected (Figure 3D). The final measured volume of the contrast agent-enhancing regions before reaching the defined experimental endpoints (as outlined in Supplementary Table S1) were in the range of 65 mm^3^ to 135 mm^3^ in four of the five included animals (see Figure 3B). Of note, calculated tumor volumes represented up to 40% of the whole brain volume in the final stage of the disease (see Supplementary Figure S2A). As visualized in Figure 3D, [^18^F]FET uptake was in good accordance with gadolinium uptake in the late phase of tumor progression at day 42. Of note, at 27 days, although several mice presented contrast-enhancing tumor lesions, [^18^F]FET-PET did not present any focal lesions.

We aimed at investigating the relationships between the measured imaging variables and the timepoints when the primary outcome parameter, i.e., symptom-free survival, was reached. Therefore, we correlated tumor volume, tumor [^18^F]FET uptake, and gadolinium concentration in the tumor region and the brain parenchyma adjacent to the main tumoral mass. We observed significant positive correlations between tumor volume, [^18^F]FET uptake, and gadolinium concentrations in the tumor region (*p* < 0.001). Moreover, evaluated imaging variables highly correlated with the primary outcome parameter (*p* < 0.001). Correlations are shown in supplementary Figure S3.

The dynamic analysis of the PET data per time point showed a steady retention in the brain up to day 27 but no tumor differentiation. Unfortunately, we could not perform FET-PET acquisitions between days 27 and 42. After 42 days, the tumors displayed an increased uptake in comparison to brain background, without wash-out dynamics, only constant increased retention (Figure 3C). In summary, the tumors were first identifiable on day 25 using gadolinium-enhanced MRI. We detected colocalizing gadolinium and [^18^F]FET enhancement on the last imaging acquisition day (day 42 after DF-1 cell implantation).

### 3.3. PDGFB-Driven Glioma Show High Basal Infiltration of Immune Cells in Comparison with the Orthotopic Syngeneic SMA560/VM-Dk Glioma Mouse Model

Treatment-naïve human glioblastomas harbor an immunosuppressive microenvironment with low numbers of infiltrating T cells and tremendous amounts of tumor-associated macrophages (TAMs) [63,64]. Current approaches in immunotherapy, e.g., by personalized peptide vaccination, can lead to an increased infiltration of CD8-positive T cells and increased immunogenicity [65]. Moreover, systemic immunosuppressive and inflammation markers can be influenced by novel therapeutic options as well [66,67,68,69].

Therefore, we assessed the composition of the glioma-associated microenvironment of treatment-naïve PDGFB-driven glioma in mice. We performed an immunohistochemical analysis of 14 markers on tumor tissue (Figure 4 and Supplementary Figure S4). We investigated the infiltration of tumor tissue by host T cells and microglia/macrophages using the well-established markers CD3, CD4, CD8 (as illustrated in Figure 4, column 3–5), CD11b, CD204, and CD163 (column 6–8).

Moreover, we compared the staining patterns with the untreated tissue of the orthotopic SMA560/VM-Dk glioma mouse model (see Figure 4, row 1) [57]. T cell-specific markers showed a stable presence in animal cohorts (both with and without imaging), and the strongest signal was detectable against CD3-positive cells. Of note, even CD8-positive cells could be often clearly distinguished, whereas in the SMA560/VM-DK, only single positive stained cells could be observed (see Figure 4, column 5). Interestingly, CD11b showed a strong staining signal in both glioma mouse models. In contrast CD204, which was widely expressed in the syngeneic SMA560/VM-Dk model, showed a reduced frequency in the evaluated PDGFB-driven glioma. Inversely, we detected an increased CD163 staining signal in the evaluated RCAS-tva model in comparison to the SMA560/VM-Dk model. Additionally, the PD1/PD-L1 axis immunosuppressive markers were assessed (as illustrated in Supplementary Figure S4). PD1 as well as ligand PD-L1 were present in evaluated tissue samples. Additional histological markers against several subtypes of B and T cells revealed comparable results to the SMA560/VM-Dk model, with a slight tendency towards decreased numbers of natural cytotoxicity, triggering receptor 1 (NCRI)-expressing cells (Supplementary Figure S4, column 3) and an increased signal for CD20-expressing cells in the evaluated tumor tissue from mice after DF-1 cell implantation (Supplementary Figure S4, column 2).

## 4. Discussion

Robust and flexible systems to precisely implement genetic and metabolic alterations in immunocompetent rodent glioma models are essential for the preclinical evaluation of therapeutic strategies [38]. Here, we aimed at further characterizing the PDGFB-driven glioma model using the RCAS-tva delivery system, monitoring tumor growth dynamics as well as tumoral metabolic activity by neuroimaging.

Genetic alterations, such as PDGFB amplification, the homozygous loss of Cyclin Dependent Kinase Inhibitor 2A (CDKN2A), and coding for p16INK4A and p14ARF, show high frequency in human glioma [4]. In general, as outlined in Figure 1 and Figure 2A, we observed robust tumor induction in a reliable manner [41,42], i.e., stable tumor formation and comparable median symptom-free survival, in two independently conducted experiments using the RCAS-tva system with a PDGFB overexpression in Cdkn2a deleted mice, underlining the high reproducibility of the model (Figure 3A). Of note, our evaluated median symptom-free survival was comparable to Hambardzumyan et al., who primarily evaluated the tumor-formation capacity of RCAS-PDGFB in transgenic mice depending on the cerebral implantation site [46]. Control groups of treatment studies, e.g., focusing on colony stimulating factor 1 (CSF1R) inhibition, have also reported similar symptom-free survival [49]. The morphological analysis of post-mortem tumor tissue revealed histological features of human glioblastoma (Figure 2C) [20]. We detected a high grade of neovascularization and necrosis areas as well as infiltrating growth behavior (Figure 2C)). Similar findings were described by Connolly et al., who established a PDGFB-driven glioma model in transgenic rats using the RCAS/tva system [70].

The multimodal neuroradiological assessment of treatment responses using classical and functional imaging modalities, evaluating tumoral growth kinetics, are essential for brain tumor surveillance [7]. Executed longitudinal MRI imaging of the PDGFB-driven glioma model revealed exponential growth kinetics of contrast-enhanced lesions (Figure 3B). Interestingly, the evaluated tumors showed comparable radiological features with the clinical situation. The time points of the first-detectable contrast-enhancing lesions and tumor volumes before reaching the experimental endpoint differed in the range of five to ten days between animals within the experimental group (Figure 3B). Despite the different onset of detectable tumoral lesions, we could observe similar growth patterns and stable median-symptom-free survival, as shown in Figure 3A in our experimental group. These findings correlate with other preclinical studies that evaluated treatment responses by MRI. The exponential growth dynamics were displayed in control groups or in tumors that acquired therapeutic resistance [49,50]. Moreover, growth dynamics might recapitulate high proliferative capacities in combination with rapid neovascularization in human glioblastoma [20].

Due to the complexity of the contrast agent application in rodent models, monitoring glioma growth in therapy studies is mostly performed by assessing T2-weighted images. However, these do not always allow reliable and clear tumor delimitations (Figure 3D). T2-weighted imaging showed perifocal edema with loss of signal in center regions, often observed in cerebral micro bleedings [71]. Of note, tumor borders are rather ill-defined in T2-weighted images (Figure 3B,D). Gadolinium uptake provides a better delineation of the tumor region, similar to the clinical situation in glioma patients [72]. In fact, the observed gadolinium uptake in this study reflected other published contrast-agent uptake patterns observed in the same model during treatment that targeted myeloid-derived suppressor cells (MDSCs) [73]. Interestingly, our findings indicated a delayed contrast-agent uptake starting at day 25 until 27 post-DF-1 cell implantation (Figure 3B). This finding entails a disruption of the BBB that produced a focal observable lesion at day 25. Although, histologically, tumor cells are present at early time points, they appear to present insufficient tissue alterations to disrupt the BBB. A late onset of BBB disruption and gadolinium-enhanced tumor visualization starting between days 25 and 27 emphasizes that appropriate therapy starting points in treatment studies are imperative for evaluating PDGFB-driven glioma. Fixed and early treatment schedules might result in the early therapy initiation of small-size lesions that poorly recapitulate the clinical situation. Accordingly, it might lead to the exaggerated interpretation of treatment responses in rodent models. Therefore, imaging-based therapy start points referring to a predefined minimum tumor volume might be helpful to increase the translational impact, i.e., comparability between in vivo modelling and human glioma patients. This consideration was implemented in several studies tackling TAMs-centered therapies. Therapy started only with a tumor volume of 40 mm^3^ in T2-weighted images, largely observed between weeks 4 and 5 after the tumor induction of PDGFB-driven glioma [49,50,74]. In some studies, group randomization referring to initially measured tumor volume was possible in preclinical rodent therapy studies, probably leading to better balanced treatment groups [49,73]. We suggest the following experimental sequence for preclinical therapeutic assessments: cell implantation, baseline imaging and determination of tumor volume, start of therapy with comparable tumor volume in all experimental groups, and clinical and imaging-based monitoring.

Longitudinal metabolic imaging using [^18^F]FET-PET detecting temporal and spatial alterations in human glioma usually encompasses the period from the initial diagnosis of a clinically apparent tumor to its evolution under therapy. In contrast, the used PDGFB-driven glioma model offers the unique possibility to monitor the interval from tumor induction to the establishment of a clinically and biologically advanced tumor by close-meshed [^18^F]FET PET imaging time points (Figure 2B and Figure 3). Longitudinal [^18^F] FET-PET imaging showed the late onset of intense and diffuse tracer uptake in tumoral regions above the background brain activity. The tumor [^18^F]FET uptake presented a similar pattern as observed post-contrast-enhancement T1-weighted images (Figure 3C,D). Taking these two patterns together, the model presents typical human PET/MRI features that are highly relevant for the diagnostic and clinical management of glioma [33]. Interestingly, [^18^F]FET-PET did not correlate with BBB permeability at early tumor stages (Figure 3C). This finding is in agreement with previously published glioma rat data, where BBB permeability did not always correlate to [^18^F]FET uptake [75]. Possible explanations for this phenomenon might be a delayed availability of amino-acid transporters in the tumor, the partial volume effect, or sensitivity limitations of PET [76]. In addition, the lack of a “washout” dynamic curve in all evaluated tumors is reminiscent of the IDH-mutant tumor in humans [77]. Therefore, the uptake behavior of wash-out negative glioblastomas and their biological mechanisms can be further investigated using this mouse model.

Next, we investigated the composition of the glioma-associated microenvironment by immunohistochemistry [63]. We detected several subtypes of infiltrating lymphocytes, as well as CD163- and CD11b-positive cells, highly suggestive of the presence of TAMs inside the glioma-associated microenvironment (Figure 4 and Supplementary Figure S4). The presence of stained TAM markers has been linked to a worse prognosis in molecular glioblastoma subtypes, and an increased frequency correlates with the respective WHO grade [64,78,79]. Additionally, the comparison of the widely used syngeneic SMA560/VM-Dk model showed similarities and differences regarding the composition of the glioma-associated microenvironment, indicating a rodent model-specific microenvironmental structure and reflecting the different genetic landscapes and immune escape mechanisms of modelled human gliomas (Figure 4) [57]. However, potential cofounding factors, such as the difference in tumor size or the experimental setup, might be considered as well. In general, glioma models using the RCAS-tva system might better reflect the natural course of disease than chemically induced or spontaneous-occurring syngeneic orthotopic glioma transplantation models, e.g., implanting GL261 cells in C57BL/6 mice, regarding tumor initiation and disease progression [80].

The PDGFB-driven model was widely used in studies with novel TAM-centered therapeutic options. For example, one study demonstrated that primarily achieved re-education of TAMs through CSF1R inhibition often led to acquired therapeutic resistance via alternative insulin growth factor 1 (IGF1)/IGFR signaling. Combination therapeutic regimes targeting the acquired resistance mechanism led to a survival benefit, underscoring the PDGFB-driven mouse model applicability towards combination therapeutic regimes [50]. Moreover, valuable insights into the dynamic composition of the tumor microenvironment under therapy and during disease progression could be achieved in a study combining two-photon microscopy and MRI measurements [74]. Even more so, the discrimination of undiscovered potential drivers of gliomagenesis can be addressed by the establishment of a novel genetic forward screen using the retroviral integration capacity of the RCAS virus for detecting potential novel oncogenes in the PDGFB-driven glioma model [81]. 

Taken together, we provided the multiparametric profiling of a PDGFB-driven glioma mouse model using the RCAS-tva delivery system and demonstrated radiological, histological, and metabolic features that are comparable to human high-grade glioma. Still, the small number of imaged animals as well as limited FET tracer availability during day 27 and 42 are a study limitation. Therefore, future close-meshed imaging studies using [^18^F]FET PET should begin close to day 25 p.i. in order to capture early model-specific tumor features.

## 5. Conclusions

Our study provided a multilayered profiling of a PDGFB-driven glioma mouse model using the RCAS-tva delivery system and discovered radiological, histological, and metabolic features that are comparable to human high-grade glioma. We conclude that our results further highlighted the translational capacities of this innovative preclinical model by reflecting relevant glioblastoma-like imaging and histological characteristics. Furthermore, future translational studies using this preclinical model might be further facilitated and reproducible by using the following experimental sequence: cell implantation, baseline imaging and determination of tumor volume, start of therapy with comparable tumor volume in all experimental groups, and clinical and imaging-based monitoring. This might optimize the design of future preclinical studies and comparability with the clinical setting.

## Data Availability

The data presented in this study are available in this article.

## Supplementary Materials

The following supporting information can be downloaded at: https://www.mdpi.com/article/10.3390/brainsci12111426/s1, Figure S1: Representative H&E images of SMA560 glioma, Figure S2: Selected immunohistochemistry staining in SMA560 and PDGFB-driven glioma, Figure S3: Longitudinal MRI dynamics and correlation with [^18^F]FET uptake, Figure S4: Correlation matrix of all imaging parameters to the primary outcome parameter, Table S1: Parameter for scoring of the experimental animals.
