# Supplementary material for: Multiparametric Longitudinal Profiling of RCAS-tva-Induced PDGFB-Driven Experimental Glioma
Source: Brain Sci. 2022 Oct 24;12(11):1426. doi: 10.3390/brainsci12111426 (PMC9688186; doi:10.3390/brainsci12111426)
Supplement: Supplementary file 1 [file brainsci-12-01426-s001.zip › brainsci-1923193-supplementary.pdf]

**Supplementary information**

**Supplementary Table S1: Parameter for scoring of the experimental animals.**

| Parameter | Phenotype | Score |
|---|---|---|
| **General appearance** | Clean skin and orifices, no pain, no weight loss | 0 |
| | Slight eye or nose discharge, slight pain, up to 10% weight loss | 1 |
| | Sticky eyes, moderate pain, up to 19% weight loss | 2 |
| | Cramps, dehydration, strong pain, max. 20% weight loss | 3 |
| **Behavior and motion activity** | Normal spontaneous-explorative behavior, normal activity | 0 |
| | Reduced spontaneous-explorative behavior, reduced activity | 1 |
| | Strongly reduced spontaneous-explorative behavior, strongly reduced activity | 2 |
| | Total inactivity | 3 |
| **Posture, facial expression and assessment of pain with the "grimace score" [1]** | Normal posture, normal facial expression | 0 |
| | Slightly hunched back, less than 5 facial attributes with score 1, little pain | 1 |
| | Moderately hunched back, grimace score: all facial attributes are moderate, moderate pain | 2 |
| | Strongly hunched back, grimace score: all facial attributes are severe, severe pain | 3 |
| **Neurological symptoms** (Behavior in the cage and on the grid, left paw paralysis) | None | 0 |
| | Slight loss-of-balance, occasionally missed steps, slight paralysis | 1 |
| | Moderate loss-of-balance, every third step missed, moderate paralysis | 2 |
| | Strong loss-of-balance, total inactivity, strong paralysis | 3 |

Compare [1].

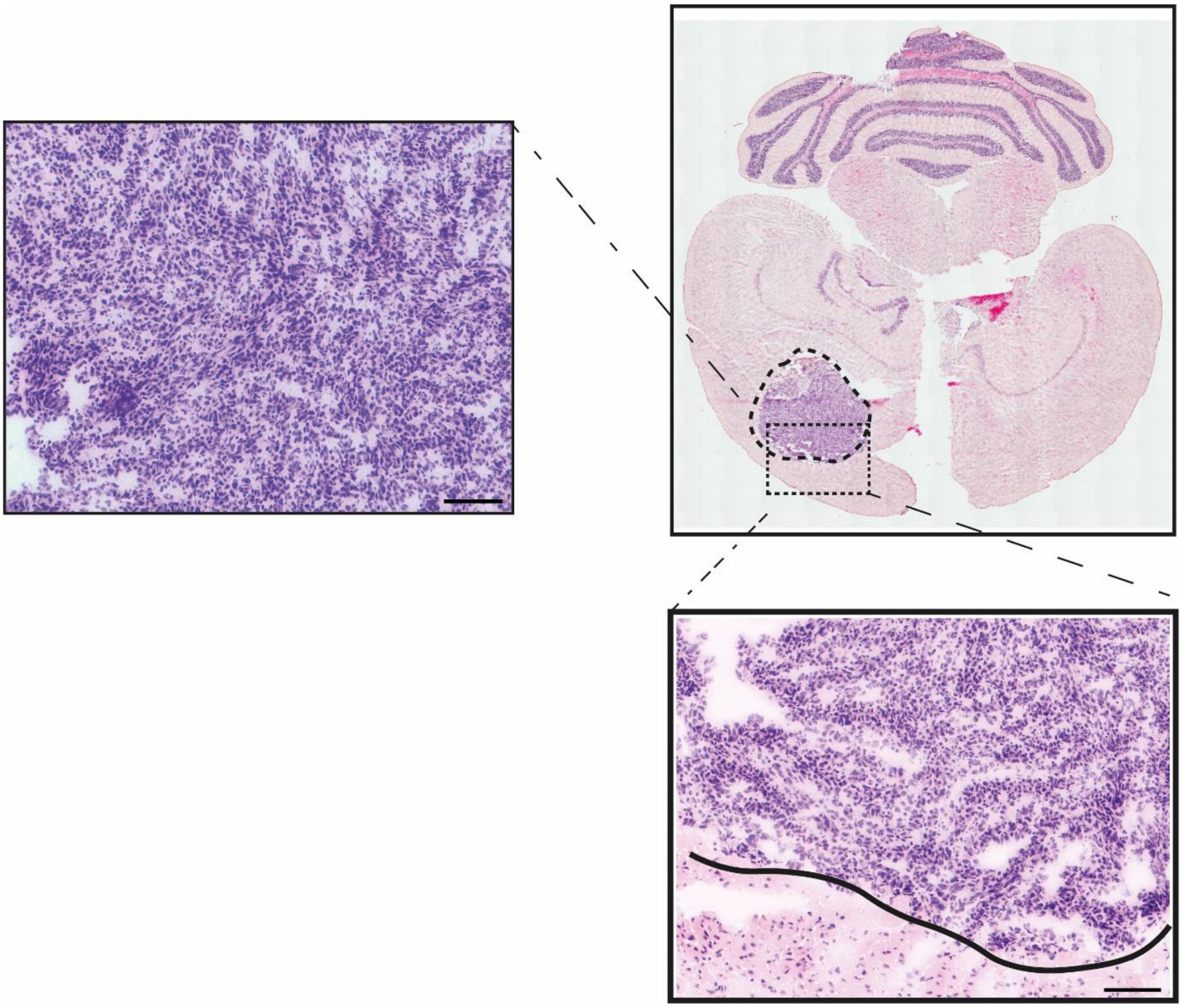

**Supplementary Figure S1. Representative H&E images of SMA560 glioma.**
Representative H&E staining of SMA560 glioma on day 18 after orthotopic tumor cell implantation into the brain of syngeneic VM/Dk mice. Morphologic features include infiltrating growth, neovascularization, and high cellular density. The black line highlights the tumor margin and the infiltration zone. Scale bars are 100 μm.

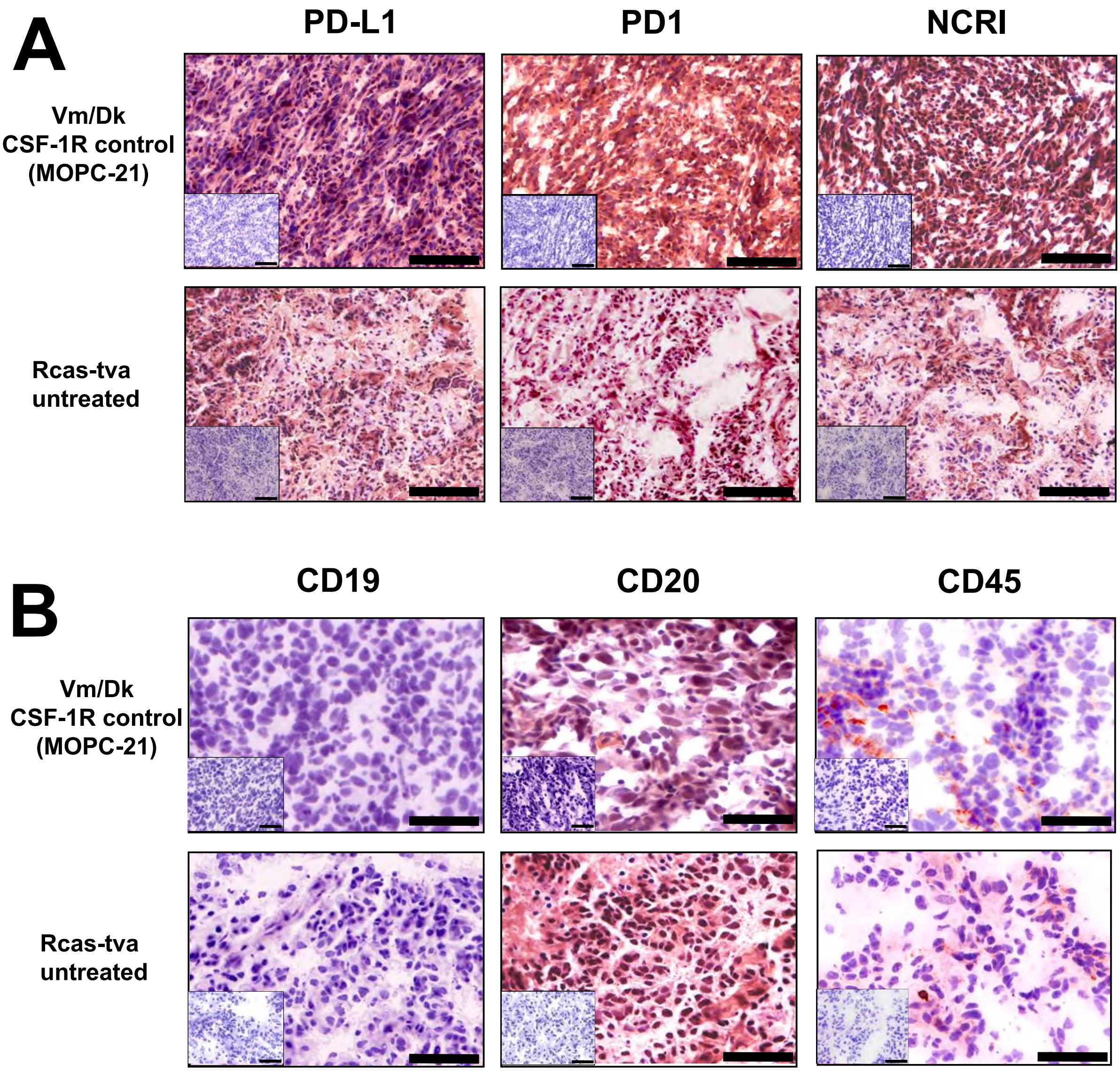


**Supplementary Figure S2. Selected immunohistochemistry staining in SMA560 and PDGFB-driven glioma.**

(**A**) Immunohistochemical staining of the PD1/PD-L1 axis and (**B**) cell markers in SMA560 glioma and PDGFB-driven glioma using the RCAS-tva delivery system (small inserts show isotype controls). Scale bars are (**A**) 100 µm in (**B**) 50µm.

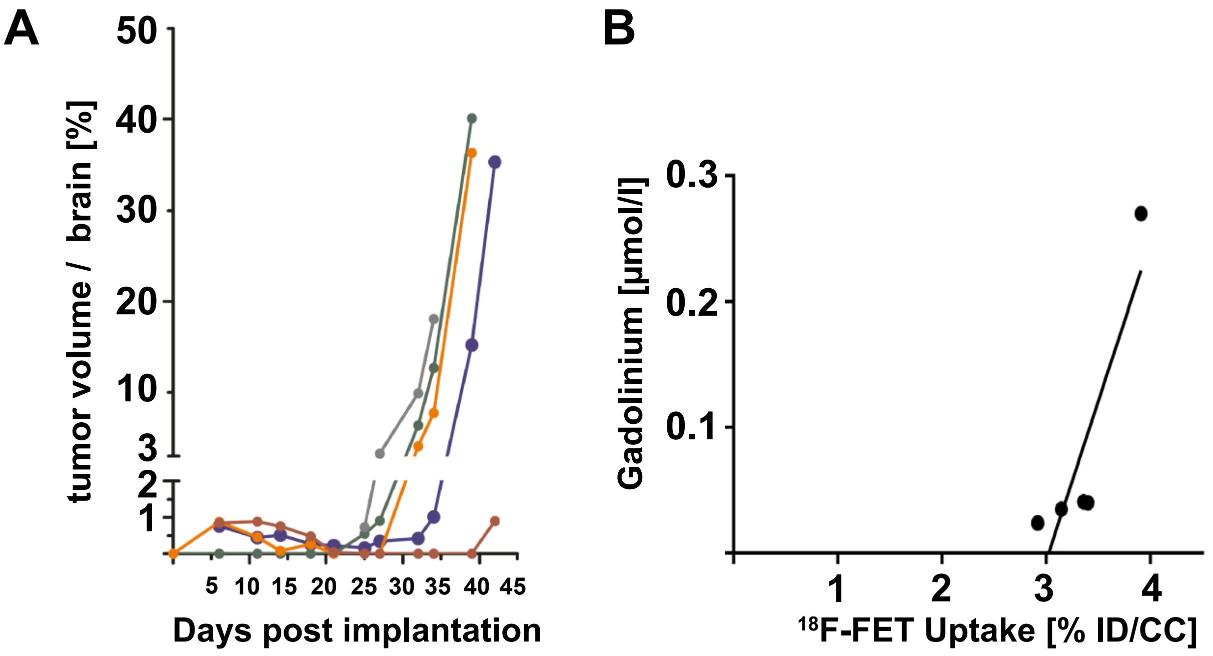


**Supplementary Figure S3: Longitudinal MRI dynamics and correlation with [$^{18}$F]FET uptake**

(**A**) Longitudinal tumor volume curve; values on y axis indicate the relative tumor volume within the whole brain (in percentage). (**B**) Correlation of gadolinium to [$^{18}$F]-FET uptake at 42 days post-implantation.

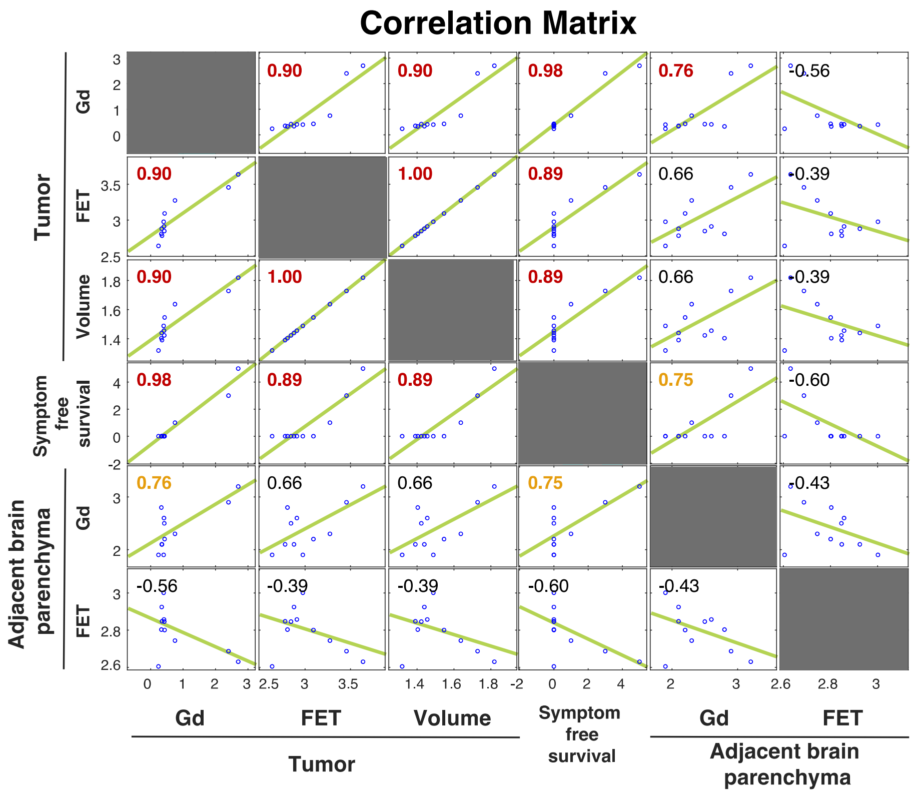


**Supplementary Figure S4: Correlation matrix of all imaging parameters to the primary outcome parameter**

Pearson correlations showed a correlation of tumor volume, tumor FET uptake, and tumor Gd uptake. These imaging parameters also correlated with the timepoints when animals reached the primary outcome parameter (symptom-free survival). (Pearson correlations are shown per graph on the top left corner, with red symbolizing a significant correlation under $p < 0.001$ and orange a $P$-value between 0.01 and 0.001).

## References

1. Langford, D.J.; Bailey, A.L.; Chanda, M.L.; Clarke, S.E.; Drummond, T.E.; Echols, S.; Glick, S.; Ingrao, J.; Klassen-Ross, T.; Lacroix-Fralish, M.L.; et al. Coding of facial expressions of pain in the laboratory mouse. *Nat. Methods*. **2010**, *7*, 447–449. https://doi.org/10.1038/nmeth.1455
